# RNA-seq studies using wheat *PHYTOCHROME B* and *PHYTOCHROME C* mutants reveal shared and specific functions in the regulation of flowering and shade-avoidance pathways

**DOI:** 10.1186/s12870-016-0831-3

**Published:** 2016-06-21

**Authors:** Stephen Pearce, Nestor Kippes, Andrew Chen, Juan Manuel Debernardi, Jorge Dubcovsky

**Affiliations:** Department of Plant Sciences, University of California, Davis, CA 95616 USA; Present Address: Department of Soil and Crop Sciences, Colorado State University, Fort Collins, CO 80523 USA; Howard Hughes Medical Institute, Chevy Chase, MD 20815 USA

**Keywords:** Wheat, *PHYB*, *PHYC*, Photomorphogenesis, RNA-seq, Shade-avoidance, Flowering

## Abstract

**Background:**

In cereal crops such as wheat, an optimal timing of developmental transitions is required to maximize grain yield. Many of these developmental changes are precisely regulated by changes in the duration, intensity or quality of light. Phytochromes are dimeric photoreceptors that absorb light maximally in the red and far-red wavelengths and induce large-scale transcriptional changes in response to variation in light quality. In wheat, *PHYC* is required for early flowering under long days. However, it is currently unknown whether this function requires the presence of *PHYB*. In this study, we characterized the role of *PHYB* in wheat development and used RNA-seq to analyze and compare the transcriptomes of *phyB*-null and *phyC-*null TILLING mutants.

**Results:**

Under long-day photoperiods, *phyB-*null plants exhibit a severe delay in flowering comparable to the delay observed in *phyC*-null plants. These results demonstrate that both genes are required for the induction of wheat flowering under long days. Using replicated RNA-seq studies we identified 82 genes that are significantly up or down regulated in both the *phyB-*null and *phyC-*null mutant relative to their respective wild-type controls. Among these genes are several well-characterized positive regulators of flowering, including *PPD1, FT1* and *VRN1*. Eight-fold more genes were differentially regulated only in the *phyB*-null mutant (2202) than only in the *phyC-*null mutant (261). The *PHYB-*regulated genes were enriched in components of the auxin, gibberellin and brassinosteroid biosynthesis and signaling pathways, and in transcription factors with putative roles in regulating vegetative development and shade-avoidance responses. Several genes involved in abiotic stress tolerance pathways were also found to be regulated by *PHYB*.

**Conclusions:**

*PHYB* and *PHYC* are both required for the photoperiodic induction of wheat flowering, whereas *PHYB* alone regulates a large number of genes involved in hormone biosynthesis and signaling, shade-avoidance response, and abiotic stress tolerance. Our analysis provides a comprehensive overview of the PHYB- and PHYC-mediated transcriptional changes during light signaling, and an initial step towards the dissection of this regulatory gene network in wheat. This further dissection will be required to explore the individual phytochrome-mediated developmental responses and to evaluate their potential to improve wheat adaptation to changing environments.

**Electronic supplementary material:**

The online version of this article (doi:10.1186/s12870-016-0831-3) contains supplementary material, which is available to authorized users.

## Background

Plants utilize external cues to precisely coordinate their growth and development with environmental conditions that maximize reproductive success. In cereal crops such as wheat, this has a direct impact on grain production, so understanding the regulatory mechanisms underlying these responses has important practical implications.

Plants are exquisitely adapted to detect variation in the quality, intensity and duration of light signals, and in response undergo rapid and dynamic transcriptional changes. These responses are mediated by several classes of photoreceptors, which absorb light of different wavelengths [1]. Among the photoreceptor families, the phytochromes absorb light maximally in the red (R) and far-red (FR) spectrum and modulate several important biological processes, including seed germination, flowering development, circadian rhythms and shade-avoidance [2, 3].

The phytochrome protein consists of two modules, both of which are essential for light signaling. The N-terminal photosensory core module is required for chromophore binding and photoconvertibility and is comprised of three domains: PAS (named from homology to PERIOD, ARYL HYDROCARBON RECEPTOR NUCLEAR TRANSPORTER and SINGLE MINDED), GAF (cGMP phosphodiesterase/adenylate cyclase/FhlA) and PHY (phytochrome-specific domains) [3]. The C-terminal module is comprised of two tandem PAS domains and a histidine kinase-like domain and is required for downstream regulatory function [4].

Phytochrome proteins can have one of two interchangeable isomeric forms; the biologically inactive, R light-absorbing Pr form and the biologically active, FR light-absorbing Pfr form [3]. Phytochromes are synthesized in the Pr form in the cytosol and, upon absorption of R light, undergo rapid conformational change to the active Pfr form, which results in their import into the nucleus [5]. In darkness or upon absorption of FR light, phytochromes in the Pfr state revert to the inactive Pr state [3]. In the nucleus, phytochrome dimers interact with a small subset of basic helix-loop-helix (bHLH) transcription factors known as PHYTOCHROME INTERACTING FACTORS (PIFs) [6]. The PIFs directly regulate a set of downstream targets by binding to conserved ‘G-box’ elements in their promoters [7]. The PIF primary targets then activate an array of secondary responses, including other transcription factors and regulators of growth and development [8]. The interaction between phytochromes and PIFs triggers the rapid multi-site phosphorylation of the latter, tagging them for degradation by the 26S proteasome machinery [9]. Phytochromes in the Pfr state inhibit the regulatory activity of PIF proteins by releasing them from their DNA targets [10, 11]. The PHY-PIF interaction also induces the degradation of the PHY protein, as part of a feedback regulatory mechanism to control active PHY levels [12]. This flexible mechanism allows for the precise adjustment of plant growth and development to subtle variations in light quality.

The phytochromes are encoded by three main clades of genes; *PHYA*, *PHYB* and *PHYC* [13]. While in the dicot lineage, gene duplication events within the *PHYB* clade have given rise to the *PHYD* and *PHYE* genes, the genomes of most monocot species, including wheat and barley, contain a single copy of each of the three phytochrome genes [13]. In Arabidopsis, a series of *phy* null mutants have been used to characterize the distinct and overlapping roles played by each phytochrome during development [2]. *PHYA* is the predominant phytochrome in seedling photomorphogenesis and regulates hypocotyl elongation during de-etiolation and the response to low fluence light [14]. *PHYB*, partially redundantly with the related *PHYD* and *PHYE* genes, regulates vegetative development, including the shade-avoidance syndrome, a response characterized by changes in plant architecture and growth under low ratios of R light to FR light (R/FR) to avoid shading by surrounding vegetation [15]. *PHYC* plays a more limited role and regulates a variety of photomorphogenesis responses throughout development in combination with other phytochromes. In both Arabidopsis and rice, PHYC activity is dependent on a functional PHYB protein [16–18]. However, in wheat, the PHYC protein is stable in the absence of other phytochromes and is sufficient to induce photomorphogenic changes when introduced into an Arabidopsis plant lacking functional endogenous phytochromes [19].

In the long-day (LD) grasses, such as wheat, barley and *Brachypodium*, *PHYC* plays a critical role in the acceleration of flowering under inductive LD conditions [19–21]. In these species PHYC is essential for the light activation of the *PHOTOPERIOD 1* gene (*PPD1 = PSEUDO RESPONSE REGULATOR37, PRR37*) [19–21], which is responsible for most of the natural variation in photoperiodic response in the temperate cereals [22–25]. Under LD, *PPD1* upregulates *FLOWERING LOCUS T1* (*FT1*), which encodes a mobile protein with homology to Phosphatidylethanolamine-Binding Proteins (PEBPs) domain [26]. The FT1 protein is transported through the phloem from the leaves to the shoot apical meristem, where it is assembled into a hexameric protein complex that directly activates the expression of the meristem identity gene *VRN1* [27, 28].

Wheat *phyC-*null mutant plants flower more than 100 days later than the wild-type control and this delay is associated with the downregulation of both *PPD1* and *FT1* [19]. The delay in flowering in the *phyC-*null mutant is more severe than the effect observed in either *ppd1-*null [29] or *ft1-*null [30] mutants, suggesting that in addition to the *PPD1-FT1*-mediated effect on flowering, *PHYC* also regulates other floral activation pathways. These additional effects on flowering time might be associated with the transcriptional changes observed in several components of the circadian clock in the *phyC-*null mutant [19].

Protein interaction studies demonstrated that the wheat PHYC protein can form both PHYC-PHYC homodimers and PHYB-PHYC heterodimers [19], but it is currently unknown whether PHYB is also necessary for the LD induction of flowering in wheat. In this study we show that the *phyB-*null mutant flowers even later than the *phyC-*null mutant, suggesting that both phytochromes are critical for flowering induction in wheat. We also describe morphological differences in the vegetative phenotype of the two mutants and characterize the subsets of genes regulated by each phytochrome using replicated RNA-seq studies. We show that both PHYB and PHYC are required for the induction of several flowering genes, and that more genes are differentially regulated only in the *phyB-*null mutant than only in the *phyC-*null mutant. *PHYB*-regulated targets include multiple genes involved in vegetative development, hormone biosynthesis and signaling, the shade-avoidance response, and abiotic stress tolerance. Our analysis provides insight into the downstream regulatory networks controlled by wheat PHYB and PHYC and identifies additional targets to further dissect light-mediated developmental signals in wheat.

## Results

### Characterization of the *phyB*-null mutant

**Fig. 1:**
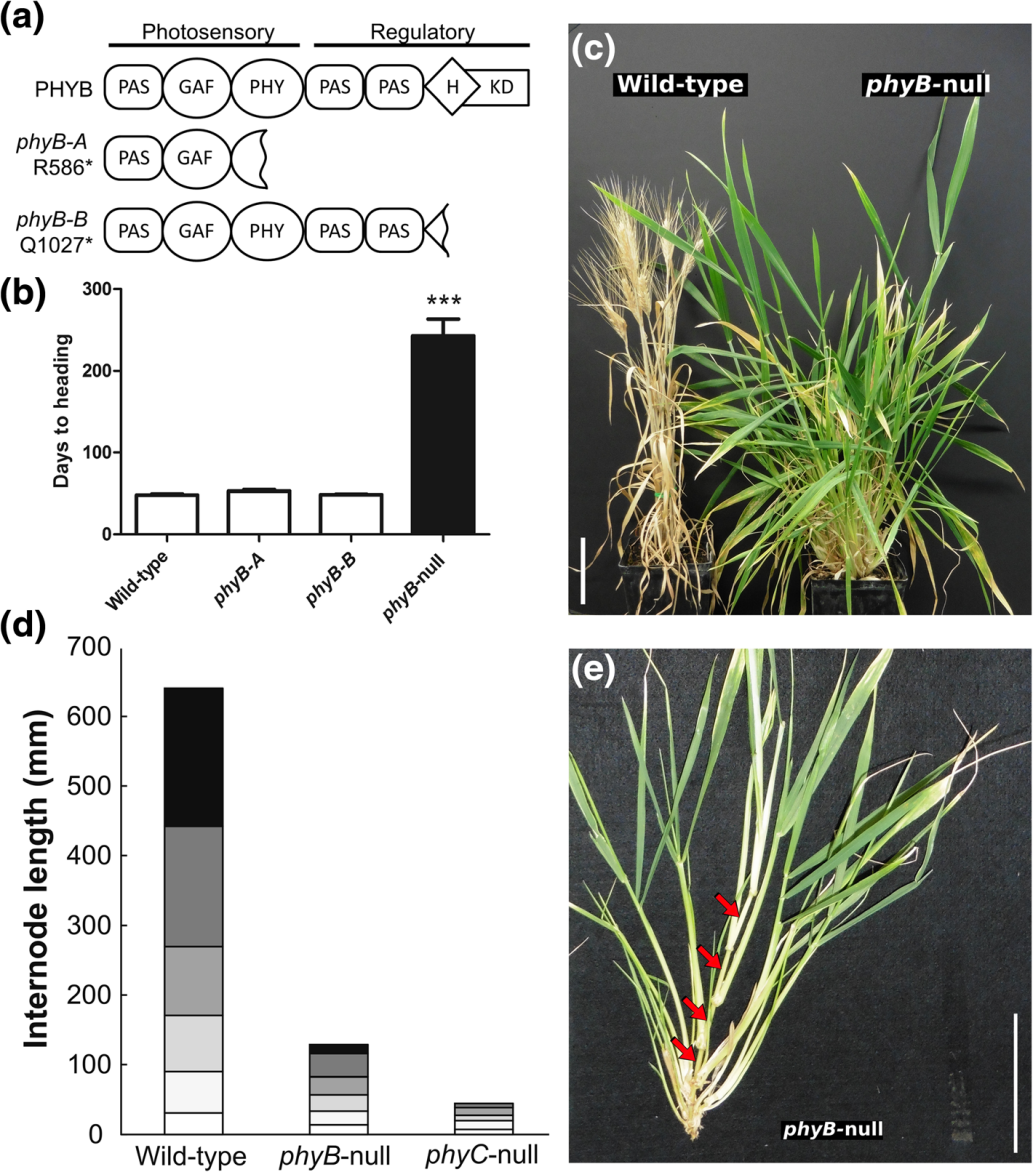
Characterization of the *phyB*-null mutant. **a** Schematic representation of the conserved functional domains of the PHYB protein and selected *phyB-A* and *phyB-B* TILLING mutants. **b** Flowering time of *phyB*-null mutant plants under LD photoperiod. Data represent the mean of at least five plants (*** *P* < 0.001). **c** Wild type and *phyB-*null mutant plants 60-days after sowing. **d** Stem length at 60 days. Individual internodes are indicated by different shades. White bars indicate the oldest internode and black bars the youngest internode. **e** Detail of a tiller from a *phyB-*null mutant plant 60-days after sowing showing elongated internodes (red arrows). Bar = 10 cm

Using a Targeting Induced Local Lesions in Genomes (TILLING) population of the tetraploid wheat variety Kronos we identified 206 mutations in the coding regions of the A and B genome copies of *PHYB* (henceforth *PHYB-A* and *PHYB-B*, respectively). Among these mutations, we selected line T4-2711 carrying a C to T change at nucleotide 1756 in *PHYB-A*. This *phyB-A* mutation generates a premature stop codon at position 586 (R586*) and a deletion of the last 641 amino acids including the entire regulatory module (Fig. 1a). For the *PHYB-B* gene, we selected line T4-2078 carrying a C to T change at nucleotide 3079. This *phyB-B* mutation generates a premature stop codon at position 1027 (Q1027*) that results in a C-terminally truncated protein lacking the distal 140 amino acids, including the histidine kinase domain (Fig. 1a). Since these C-terminal domains are required for phytochrome signaling [4], there is a high probability that both selected mutant lines encode non-functional PHYB proteins, and are thus loss-of-function mutations. Both mutants were backcrossed twice to wild-type Kronos to reduce the background mutation load, and were then intercrossed to select a plant homozygous for both mutations (*phyB-A*/*phyB-B*), hereafter referred to as *phyB-*null. We confirmed the presence of these mutations in the cDNA of each mutant line using RT-PCR and Sanger sequencing.

Under LD conditions, neither the *phyB-A* (4.7 days delay, *P* >0.05) nor *phyB-B* (0.3 days delay, *P* >0.05) single mutants exhibited significant changes in flowering time when compared to wild-type sister lines (Fig. 1b). However, the *phyB-*null mutant (lacking any functional *PHYB* gene) exhibited a severe delay in flowering of 195 days (*P* <0.001, Fig. 1b). Furthermore, the emerged spikes did not set seeds, despite the formation of all constituent parts of the spikelet and floral organs (Additional file 1: Figure S1). This significant delay in flowering is even more severe than the large delay previously observed in the *phyC-*null mutant (108-days delay in flowering [19]) and demonstrates that both *PHYB* and *PHYC* genes are required for the induction of wheat flowering under LD.

The late flowering phenotype of the *phyB*-null mutant was associated with an extended vegetative developmental phase and other differences in the plant’s morphology (Fig. 1c). The rate of leaf emergence in *phyB*-null mutants was significantly faster (average 3.3 days per leaf) than in either the wild-type control plants (3.7 days per leaf, *P* <0.001) or the *phyC*-null mutant (3.5 days per leaf, *P* <0.05, Additional file 1: Figure S2a). We measured the size of the most recently expanded leaf at three different timepoints (36, 43 and 50 days). At each stage, leaves of the *phyB*-null mutant plant were significantly longer (*P* <0.05) than the wild-type control (Additional file 1: Figure S2b). Leaves in the *phyB*-null plant were also wider, on average, than the wild-type control, although these differences were significant only at 36 days (*P* <0.05, Additional file 1: Figure S2c). By comparison, the *phyC*-null mutant showed significantly longer (*P* <0.001, Additional file 1: Figure S2b) and narrower leaves (*P* <0.05, Additional file 1: Figure S2c) than the wild-type at all three timepoints. We also measured stem length at 60 days of age, at which stage the wild-type plant had initiated flowering development, associated with rapid stem elongation (Fig. 1d). As expected for plants in the vegetative stage, both *phyB*-null and *phyC*-null mutants exhibited greatly reduced stem elongation when compared to the wild-type, although the total number of internodes was unaffected in both mutants. Interestingly, the internodes in the *phyB*-null mutant were significantly (*P* <0.01) longer than the *phyC*-null mutant (Fig. 1d and e, Additional file 1: Figure S1e). Finally, we compared germination rates between *phyB-*null and wild-type controls and found no significant differences.

Taken together, these observations suggest that while both *PHYB* and *PHYC* genes are important during the regulation of flowering development, *PHYB* also appears to play an important role during vegetative development, influencing the rate of leaf production and cellular elongation in both stems and leaves.

Plants with mutations in only one *PHYB* homoeologue (*phyB-A* or *phyB-B*) showed no phenotypic differences with the wild-type plants, which is consistent with the lack of differences in flowering time. This last result demonstrated that the *PHYB-A* and *PHYB-B* homoeologues exhibit a large degree of functional redundancy.

### Identification of high-confidence differentially expressed genes

To analyze and compare genes regulated by *PHYB* and *PHYC* we performed replicated RNA-seq studies. Leaf tissue was harvested in the morning from LD-grown four-week-old *phyB*-null and *phyC-*null mutant plants, and from their respective wild-type sister lines, which were used as controls (Additional file 1: Figure S3). To reduce the incidence of false positives (genes incorrectly defined as differentially expressed between genotypes), we performed the complete experiment twice, using four biological replicates per genotype in each experiment. We sequenced a total of 32 RNA-seq libraries (four biological replications * four genotypes * two experiments), generating an average of 49.1 million 50 bp single-end reads per sample (Additional file 1: Table S1). We mapped 95.3 % of these reads to gene-coding regions identified within the draft assembly of the wheat genome from the International Wheat Genome Sequencing Consortium (v2.2, see Methods) [31]. All subsequent analyses were performed using only those sequencing reads that mapped uniquely to one transcribed locus (average 58.5 % of reads, Additional file 1: Table S1).

A Principal Component Analysis including the expression results for *phyB*-null, *phyC*-null and their respective controls showed a good separation among genotypes (Additional file 1: Figure S4a). The first principal component separated the *phyC-*null samples from those obtained from the wild-type sister plants. The second principal component separated the *phyB-*null samples from those corresponding to the wild-type sister plants. Limited differences were detected in these two components between the two experimental replications (Additional file 1: Figure S4a).

When each phytochrome was analyzed separately, the two *PHYB* experimental replications clustered together (Additional file 1: Figure S4b), but the two *PHYC* experimental replications clustered in two groups separated by the second principal component (Additional file 1: Figure S4c). These two *PHYC* experimental replications were performed in growth chambers of the same brand and model, and with identical halide light configurations. However, after we saw the differences in gene expression we reexamined the chambers and realized that they differed in their ballast systems, which may have caused some of the observed variation between experimental replicates. Although light intensity at R (~660 nm) and FR (~730 nm) wavelengths varied in these two chambers, the R/FR ratio was similar (2.61 in the first chamber and 2.83 in the second). Despite this variability, the first component clearly differentiated the *phyC*-null samples from the wild-type sister plants (Additional file 1: Figure S4c).

### Defining *PHYB* and *PHYC* regulated genes in wheat

**Fig. 2:**
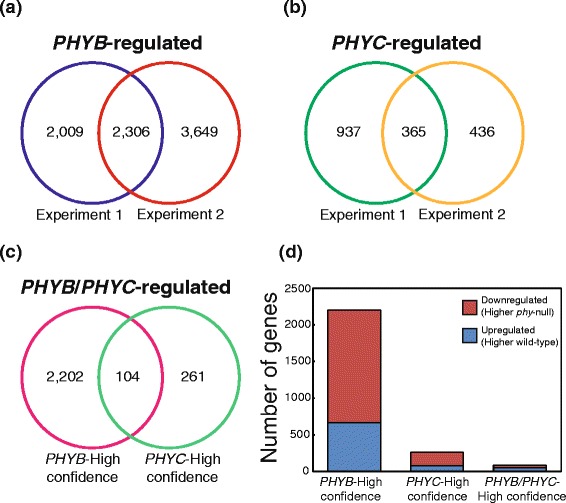
Numbers of differentially expressed genes in each experimental replicate regulated by (**a**) *PHYB*, (**b**) *PHYC* and (**c**) By both *PHYB* and *PHYC*. **d** Proportion of up- and down-regulated genes in each class

Pair-wise comparisons of gene expression values identified 2306 high-confidence PHYB-regulated genes which were differentially expressed between the *phyB*-null mutant and wild-type sister lines in the same direction in both experimental replications (4315 PHYB-regulated genes in experiment one and 5955 in experiment two at False Discovery Rate (FDR)-adj *P* <0.01, Fig. 2a). Only one gene was differentially expressed in both experimental replicates in opposite directions, and was excluded from the analysis. Because the experimental replications were performed independently, these high-confidence genes have an expected FDR < 0.0001 (<0.01 * <0.01). We also identified a further 5656 genes which were differentially expressed in just one of the two experimental replications, which are hereafter referred to as FDR-adj *P* < 0.01 PHYB-regulated genes. A similar comparison between the *phyC-*null mutant and wild-type identified 365 high-confidence PHYC-regulated genes regulated in the same direction (1302 PHYC-regulated genes in experiment one and 801 in experiment two at FDR-adj *P* <0.01, Fig. 2b) and 1373 FDR-adj *P* < 0.01 PHYC-regulated genes. Normalized expression values and FDR-adjusted *P* values for all genes are provided in Additional file 2 to facilitate the re-analysis of this data using different levels of stringency.

To limit the impact of variation between experimental replicates, and to reduce the incidence of false positives, we focused all subsequent analyses only on high-confidence PHYB and PHYC differentially-expressed genes (Additional file 3). Full details of FDR-adj *P* < 0.01 PHYB- and PHYC-regulated genes are provided in Additional file 4. Furthermore, all expression data is publicly available through “WheatExp”, an online wheat expression database and visualization tool [32].

A comparison of the 2567 high-confidence genes differentially regulated by PHYB and/or PHYC revealed 104 that were commonly regulated by both genes (Fig. 2c). Of these 104 common genes, 82 were differentially regulated in a concerted manner in the same direction (i.e., induced by both *PHYB* and *PHYC*). The remaining 22 genes were regulated in the opposite directions (i.e., induced by *PHYB* but suppressed by *PHYC* or vice versa). The number of high-confidence genes that were detected only in the *phyB*-null mutant (2202) was 8.5-fold higher than the number differentially expressed only in the *phyC*-null mutant (261, Fig. 2c). Similar proportions were identified among the 6693 FDR-adj *P* <0.01 PHY-regulated genes; 5320 were differentially expressed only in the *phyB-*null mutant, 1037 were detected only in the *phyC*-null mutant and 336 were commonly regulated in both mutants (Additional file 4). A majority of the high-confidence *PHYB-*regulated (69.9 %) and *PHYC-*regulated (70.9 %) genes exhibited higher expression levels in the respective *phy-*null mutant, suggesting that they are downregulated by the active PHY protein (Fig. 2d). In contrast, among the 82 genes regulated by both *PHYB* and *PHYC*, the majority (59.8 %) exhibited lower expression levels in the respective *phy-*null mutant, suggesting that they are upregulated by the functional PHY proteins (Fig. 2d).

### Genes with similar effects in the *phyB*-null and *phyC*-null mutants include multiple flowering regulation genes

Functional analysis of the 82 genes similarly regulated in the *phyB-*null and *phyC-*null mutants showed significant enrichment for genes with roles in transcriptional regulation (Additional file 1: Table S2). Consistent with the late-flowering phenotypes of *phyB-*null and *phyC-*null plants, both mutants showed greatly reduced transcript levels of a number of well-characterized positive regulators of flowering, including *PPD-B1*, *VERNALIZATION1, FRUITFULL2* and *FT1* (Table 1). The A-genome copy of *PPD1* is also downregulated in both *phyB-*null and *phyC-*null plants but is not among the high-confidence genes (Traes_2AS_2FCD59730, Additional file 2). The *PPD-A1b* allele in Kronos carries a deletion in the promoter region that causes altered expression levels independently of the photoperiod [24]. Our results demonstrate that this allele is expressed even in the absence of functional PHYB and PHYC phytochromes. Several other genes involved in the regulation of flowering time were also downregulated in both mutants, including *GIGANTEA* (*GI*), a circadian clock output gene, *CONSTANS9* (*CO9*), a regulator of barley flowering under SD [33], and two flowering activators belonging to the GATA-domain family of transcriptional regulators [34, 35] (Table 1).

**Table 1:** Selected flowering time genes regulated in concert by both *PHYB* and *PHYC*

		Avg. normalized counts				
Gene	Ensembl ID	*PHYB*		*PHYC*		
		Wild-type	*phyB*-null	Wild-type	*phyC-*null	Putative function
Downregulated in *phyB*-null and *phyC*-null mutants						
* PPD-B1*	Traes_2BS_8BED816B1.1	4,505	37	2,285	79	Flowering induction
* VRN-A1*	Traes_5AL_13E2DEC48.2	5,605	306	2,647	272	Meristem identity
* VRN-B1*	Traes_5BL_89636D032.1	1,573	73	884	89	Meristem identity
* FUL-A2*	Traes_2AL_20C2D79E1.2	820	2	279	3	Meristem identity
* GI-B*	contig105156	739	233	642	169	Circadian clock output
* FT-A1*	Traes_7AS_EBD5F1F54.1	590	2	331	1	Flowering induction
* FT-B1*	Traes_7BS_581AA844D.1	797	1	267	2	Flowering induction
* GATA-B1*	contig67606	69	1	77	1	Flowering induction
* GATA-A7*	td-k51_contig_84804	194	2	225	2	Flowering induction
* CO-B9*	Traes_1BL_688EF6A1A.1	594	197	410	158	Flowering induction
Upregulated in *phyB*-null and *phyC*-null mutants						
* TaAGL33-A*	Traes_5AL_6FF34F1C3.2	43	300	51	248	Flowering induction
* TaAGL33-B*	Traes_4BL_5AF7ACF03.2	5	132	6	62	Flowering induction
* CO-A1*	Traes_7AS_F46AC277B.1	19	219	14	126	Flowering induction

We also identified several flowering time genes which showed the opposite response, with significantly elevated expression levels in both the *phyB-*null and *phyC-*null mutant (Table 1). These included *CONSTANS1* (*CO1*), a gene central to flowering induction in Arabidopsis [36] and both A and B homoeologues of *AGAMOUS-LIKE 33* (*TaAGL33*) a gene homologous to *HvODDSOC2*, which delays flowering when overexpressed in barley [37] (Table 1).

To further explore the role of *PHYB* in the regulation of flowering, we selected six flowering time genes for validation by qRT-PCR and assayed their transcript levels in wild-type and *phyB*-null plants at three timepoints (two-week, four-week and six-week old plants). Four genes (*PPD1*, *FT1, VRN1* and *CO1*) were chosen from the high-confidence genes regulated by both *PHYB* and *PHYC. VRN1* and *FT1* expression was significantly higher in the wild-type than the *phyB*-null mutant at all three timepoints, *PPD1* expression was higher at the four-week and six-week timepoints and *CO1* expression was significantly reduced in wild-type plants at the six-week timepoint, confirming the role of *PHYB* in the regulation of these genes (Additional file 1: Figure S5). These four genes were shown to be regulated by *PHYC* in a previous study [19]. Two additional genes from the FDR-adj *P* < 0.01 *PHYB*-regulated genes were also selected (*FT2* and *VRN2*, Additional file 4). There were no significant differences in the expression of either of these genes between wild-type and the *phyB*-null mutant at the four-week timepoint, consistent with the RNA-seq data. However, the expression of both genes was significantly different between genotypes at six-weeks, demonstrating that *PHYB* regulates the expression of these genes at a later stage of development (Additional file 1: Figure S5).

### High-confidence genes differentially regulated only in the *phyC*-null mutant

We next examined the 261 high-confidence genes that showed a significant response in the *phyC-*null mutant, but showed no significant differential expression in the *phyB*-null mutant. Functional enrichment data showed an over-representation of genes with roles in photoperiodism and flowering time (Additional file 1: Table S2). These included *FT3,* a member of the PEBP gene family that is expressed under SD at higher levels than *FT1* and *FT2* [30] and the circadian clock gene *PRR95*, which are both downregulated in the *phyC*-null mutant (Table 2). Also included in this list was *TaAGL41*, a MADS-box gene which is a close homolog of *TaAGL33/ODDSOC2*, which was upregulated in the *phyC*-null mutant (Table 2).

**Table 2:** Selected *PHYC*-specific regulated genes

Gene	Ensembl ID	Avg. normalized counts		Putative function
		Wild-type	*phyC*-null	
Downregulated in *phyC*-null mutants				
* FT-B3*	Traes_1BL_2C43B822A.1	59	7	Flowering regulation
* PRR95-A*	Traes_5AL_852A1474C.1	1,423	456	Circadian clock
Upregulated in *phyC*-null mutants				
* TaAGL41-B*	TRAES3BF009000010CFD_t1	45	121	Flowering regulation

### High-confidence genes differentially regulated only in the *phyB*-null mutant

The genes differentially regulated only in the *phyB*-null mutant included those with putative roles in flowering, hormone biosynthesis and signaling, shade-avoidance, and abiotic stress tolerance (Table 3).

**Table 3:** Selected *PHYB-*specific regulated genes

Gene	Ensembl ID	Avg. normalized counts		
		Wild-type	*phyB*-null	Putative function
Flowering time				
* CCA-B1*	Traes_7BL_998EC9F74.2	29,787	13,084	Circadian clock output
* LHY-B*	Traes_6BL_2F2381640.1	126	43	Circadian clock output
* FT-like_chr3-A*	Traes_3AS_4310A2281.1	4	44	PEBP-family
* FT-like_chr3-B*	TRAES3BF053100340CFD_t1	2	303	PEBP-family
* FT-like_chr6-A*	Traes_6AL_66B24F155.1	21	138	PEBP-family
* FT-like_chr2-A*	Traes_2AL_2F198B97C.1	0	10	PEBP-family
* FT-like_chr5-1-A*	Traes_5AL_96274800D.1	1	95	PEBP-family
* FT-like_chr5-2-A*	Traes_5AL_EFB6E50C9.2	179	27	PEBP-family
* FPF-like-A1*	Traes_2AS_329CCD131.1	63	7,688	Flowering promotion
* FPF-like-B1*	Traes_2BS_EA4D55C79.1	35	3,503	Flowering promotion
* FPF-like-B2*	Traes_2BL_600226046.1	140	1,538	Flowering promotion
* FPF-like-A3*	Traes_6AS_447C71E5B.1	13	70	Flowering promotion
* FPF-like-A4*	Traes_5AL_6FD9AE7EB.1	91	7	Flowering promotion
* VIL-A2*	Traes_6AS_DF6C22BF3.1	1,315	539	Vernalization
* VIL-B2*	Traes_6BS_2B746261B.1	1,030	337	Vernalization
Hormone biosynthesis and signaling				
* TAR-A2*	Traes_3AS_FF5C06A87.1	26	94	Auxin biosynthesis
* TAR-B2*	td-k55_contig_11729	559	1609	Auxin biosynthesis
* IAA-A12*	td-k61_contig_15261	1	20	AUX/IAA
* IAA-B12*	Traes_5BL_9301BD154.1	41	143	AUX/IAA
* IAA-B15*	Traes_1BS_8A19C460B.1	191	440	AUX/IAA
* IAA-A16*	Traes_3AS_771897131.2	71	163	AUX/IAA
* IAA-B17*	Traes_7BL_74071485F.2	1,041	1,859	AUX/IAA
* IAA-A19*	Traes_1AL_859346448.1	380	689	AUX/IAA
* ARF-B6*	Traes_6BS_BD894AD26.1	335	88	ARF
* ARF-B9*	contig33661	16	0	ARF
* ARF-B17*	Traes_7BL_66296695F.1	2,071	493	ARF
* WAT1-like-A1*	td-k25_contig_69900	3	16	Auxin transport
* WAT1-like-B2*	Traes_5BL_CBB9D7E7F.1	552	114	Auxin transport
* PIN3-like-B*	Traes_6BL_F93C57B09.1	69	152	Auxin transport
* GA20ox-A1*	Traes_4AL_FABDF4EDA.1	77	423	GA biosynthesis
* GA20ox-B2*	td-k51_contig_72276	20	2,482	GA biosynthesis
* GA20ox-A4*	Traes_1AL_3A716350F.2	4	229	GA biosynthesis
* GA20ox-B4*	Traes_1BL_32506F819.1	8	219	GA biosynthesis
* GA2ox-A6*	Traes_2AL_BA387175F.1	2	37	GA catabolism
* GA2ox-A11*	Traes_4AS_7DC625FF5.1	3	47	GA catabolism
* GA2ox-B11*	Traes_4BL_63EFE8C91.1	1	36	GA catabolism
* ACC synthase-A*	Traes_4AL_A5B9F7B36.1	2	28	Ethylene biosynthesis
* ACC oxidase-B*	Traes_5BL_6AAC89B49.1	164	857	Ethylene biosynthesis
* BZR1-like-A*	Traes_3AS_5A6A80EA5.1	153	916	BR signaling
* BZR1-like-B*	td-k21_contig_21617	113	681	BR signaling
* PYL4-like-A1*	Traes_4AS_72BEF89AC.1	115	19	ABA signaling
* PYL4-like-B1*	Traes_4BL_E43C1BB11.1	322	80	ABA signaling
* PYL4-like-B2*	Traes_2BS_6428AA6CC.1	323	146	ABA signaling
Transcription factors				
* PIF-B3*	Traes_1BS_D1FCBFBE8.1	35	3,065	bHLH
* bHLH47-like1-A*	Traes_2AS_9AEA9BDEA.1	87	5,046	bHLH
* bHLH47-like2-A*	Traes_2AS_C4568AE60.1	606	1,740	bHLH
* PIL1-like-A*	Traes_5AL_85F3BE385.2	323	698	bHLH
* BIM-A2*	Traes_5AL_0D4BDDDCD.1	93	248	bHLH
* BIM-B2*	Traes_5BL_AAC9C7238.2	615	1,453	Bhlh
* ATHB2-A*	Traes_2AL_EF9549D16.1	24	640	Homeobox domain
* ATHB2-B*	Traes_2BL_02479C76A.1	4	332	Homebox domain
* SPL14-like-A*	Traes_7AS_FB2A769B5.1	93	182	SPL
* SPL14-like-B*	Traes_7BS_7ACA0B10A.2	36	128	SPL
Growth and cell elongation				
* XTH-like-B*	Traes_4BS_64FB912EE.1	18	103	Cell elongation
* LNG-1-like-A*	Traes_2AS_2C93BAE62.1	204	1,102	Cell elongation
* LNG-2-like-A*	td-k25_contig_4862	0	23	Cell elongation
* PROG –A1*	td-k41_contig_81185	90	502	Vegetative growth
* PROG –B1*	td-k41_contig_81185	16	173	Vegetative growth
* BSH-B*	Traes_1BS_E09101AE8.1	296	640	Vegetative growth
* GT-A1*	Traes_4AS_1EA23DE08.1	11	92	Vegetative growth
* TZP-A*	Traes_3AL_5006FA990.1	1,766	694	Architecture
* TZP-B*	isotig04736	7,439	2,774	Architecture
Cold tolerance				
* ICE-B41*	td-k35_contig_73009	103	12	*CBF* induction
* WCOR413-A*	Traes_5AL_F7649C79D.1	31	125	Cold response
* WCS19a-B*	Traes_2BL_6382E3EFF.1	1	23	Cold response
* WCOR14-B*	td-k25_contig_47404	5	208	Cold response
* WCOR15-A*	Traes_2AL_079988C38.1	18	1,025	Cold response

At the morning time point sampled in this study, the transcript levels of *LATE ELONGATED HYPOCOTYL* (*LHY*) and *CIRCADIAN CLOCK ASSOCIATED1* (*CCA1*) were significantly higher in the wild-type than in the *phyB-*null mutant.

In addition to its shared role with *PHYC* in activating *FT1* expression, *PHYB* also regulates six other uncharacterized members of the PEBP gene family independently of *PHYC* (Table 3). These six genes are distinct from the *FT-like* genes described previously in wheat [30]. Interestingly, only one of these putative *FT-like* genes (the orthologue of *HvMOTHER OF FT1 = FT-*like_chr5-2, Table 3) showed the same transcriptional response as *FT1* (downregulation in the *phyB-*null mutant), with the remainder showing significantly higher expression in the *phyB-*null mutant. *PHYB* also specifically regulates five *FLOWERING-PROMOTING FACTOR-like* (*FPF-like)* genes, four of which are significantly upregulated in the late-flowering *phyB-*null mutant (Table 3).

The wheat *VERNALIZATION INSENSITVE 3-LIKE 2* gene (*VIL2*) [38] encodes a plant homeodomain (PHD)-finger protein and was significantly downregulated in the *phyB-*null mutant (Table 3). This gene is related to the Arabidopsis *VERNALIZATION INSENSITIVE 3* gene, which plays an important role in the vernalization response in Arabidopsis (Table 3). However, the function of this gene in wheat has yet to be established [38].

*PHYB-*regulated targets exhibit an enrichment of genes with roles in the biosynthesis and signaling of several classes of hormones (Additional file 1: Table S2). Within the auxin pathway, the *phyB*-null mutant exhibited an upregulation of the auxin biosynthesis gene *TRYPTOPHAN AMINOTRANSFERASE RELATED 2* (*TAR2*) and five *AUX/IAA* genes, and a downregulation of three *AUXIN RESPONSE FACTOR* (*ARF*) genes (Table 3). Auxin signaling is modulated by the hormone’s cellular location, which is controlled by the activity of auxin transporters [39]. We identified three differentially expressed genes encoding auxin transporters, two homologous to the vacuole-localized *WALLS ARE THIN 1* (*WAT1*) and another to the plasma-membrane localized *PIN-FORMED3* (*PIN3*) (Table 3, Fig. 5).

The phenotypic changes in the *phyB-*null mutant were consistent with an increased rate of gibberellin (GA) biosynthesis, a plant hormone closely linked to phytochrome-mediated growth promotion [40] and to wheat spike development [41]. Three members of the *GA20oxidase* family, which encode enzymes catalyzing the rate-limiting reaction in GA biosynthesis, were upregulated, as were two genes encoding GA-deactivating enzymes, *GA2ox6* and *GA2ox11* (Table 3). The expression of both A and B homoeologues of *BRASSINAZOLE RESISTANT 1* (*BZR1*), a brassinosteroid-responsive transcription factor which positively regulates cell growth, were upregulated in the *phyB-*null mutant (Table 3), as were two ethylene biosynthesis genes, one encoding an ACC-synthase and another encoding an ACC-oxidase (Table 3). We also found that *phyB-*null mutant exhibited reduced expression of two putative abscisic acid (ABA) receptors of the *PYRABACTIN-RESISTANCE-LIKE* (*PYL*) gene family, indicative of a reduced sensitivity to this hormone (Table 3). Taken together, these findings indicate that *PHYB* plays a prominent role in regulating GA, BR, auxin, ABA and ethylene biosynthesis, transport and signaling pathways.

In Arabidopsis, a subset of genes has been identified which are differentially expressed in response to low R/FR and that are required for the shade-avoidance response. These genes include different classes of transcription factors, particularly members of the bHLH and homeobox leucine zipper family. We identified three bHLH genes which were significantly upregulated in the *phyB-*null mutant with high homology to characterized PIF genes (Table 3). Other bHLH genes upregulated in the *phyB-*null mutant included the wheat orthologue of *PHYTOCHROME INTERACTING LIKE PROTEIN1* (*PIL1*)*,* which regulates cell wall expansion genes during rice stem elongation [42] and *BES1-INTERACTING MYC-LIKE PROTEIN 2* (*BIM2*), a bHLH transcription factor with a role in mediating brassinosteroid signaling during shade-avoidance [43] (Table 3). We also found a significant upregulation in the *phyB-*null mutant of two homeobox leucine zipper transcription factors. The first, *ATHB2*, is a direct PIF target which regulates transcriptional responses to light quality during shade-avoidance [44], while the second, *GRASSY TILLERS 1* (*GT1*), regulates tillering and bud outgrowth in the grasses [45]. Finally, one member of the *SQUAMOSA PROMOTER BINDING-LIKE* (*SPL*) family of transcription factors with highest similarity to Arabidopsis *SPL14* was upregulated in the *phyB-*null mutant. Mutations in this gene result in plants with elongated petioles, a characteristic trait of the shade-avoidance response [46].

Several regulators of cellular growth and plant architecture were significantly upregulated in the *phyB-*null mutant (Table 3). These included a putative *XYLOGLUCAN ENDOTRANSGLUCOSYLASE/HYDROLASE* (*XTH*) gene, which encodes an enzyme that facilitates cell wall elongation to enhance cellular growth and expansion [47], *PROSTRATE GROWTH 1* (*PROG1*), a Cys_2_-His_2_ zinc-finger protein regulating plant architecture and panicle angle [48, 49]*,* two members of the *LONGIFOLIA*-like (*LNG-like*)gene family, which promote longitudinal cell elongation in Arabidopsis [50] and *BUSHYGROWTH* (*BSH*), a member of the *SUCROSE NON FERMENTING* (*SNF*) gene family which has a putative role in regulating plant architecture and seed set [51].

In Arabidopsis, *PHYB* modulates freezing tolerance in response to changes in light quality [52]. A connection between these two traits may also exist in wheat, since we found that the wheat *phyB-*null mutant exhibited a significant upregulation of four *COLD REGULATED* (*COR*) genes and downregulation of one member of the *INDUCER of CBF EXPRESSION* (*ICE*) gene family [53] (Table 3).

In addition to protein-coding genes, changes in development can be induced by non-coding parts of the genome, such as miRNAs [54]. We identified loci encoding pri-miRNAs that were specifically upregulated in either the *phyB*-null or *phyC*-null mutants (Table 4). In the *phyB-*null mutant, two homoeologous loci encoding *pri-miR530* were the most significant differentially expressed genes in our experiment. The mature miR530 is predicted to target a transcription factor with high similarity to *TANDEM ZINC KNUCKLE/PLU3* (*TZP*), which has a role in regulating plant growth and architecture downstream of the circadian clock and light signaling pathways [55]. This gene exhibits the expected inverse transcriptional response to *pri-miR530*, with significantly reduced expression in the *phyB-*null mutant (Table 4). We also found a significant upregulation in the *phyB-*null mutant of one locus encoding *pri-miR393* and another encoding *pri-miR156g* (Table 4). miR393 is predicted to target two *TIR1*-like members of auxin receptors [56–59], but the expression of these two targets was not significantly reduced in the *phyB*-null mutant (Table 4). Members of the miR156 family target multiple *SPL* genes during development [60], but we did not observe significant changes in the expression of any *SPL* genes carrying the miR156 target site at this developmental stage (Table 4).

**Table 4:** *PHY-*regulated pri-miRNAs and the predicted targets of their mature miRNA in wheat

		Pri-miRNA counts			Target counts	
Pri-miRNA	Ensembl ID	Wild-type	*phyB-*null	Putative target	Wild-type	*phyB-*null
*PHYB* targets						
* miR530-A*	isotig11608	16	7,627	*TZP*	4,603	1,734
* miR530-B*	isotig11608	12	3,518			
* miR393b-A*	td-k31_contig_70403	11	45	*TIR1-like1*	269	270
				*TIR1-like2*	469	391
* miR156g-A*	td-k31_contig_75281	34	123	*SPL* family	Multiple	Multiple
*PHYC* targets		Wild-type	*phyC-*null	Putative target	Wild-type	*phyC-*null
* miR5200-A*	td-k35_contig_61886	72	955	*FT1*	342	2
* miR5200-B*	td-k21_contig_55327	2,613	12,198			

In the *phyC-*null mutant, we identified a significant upregulation in the expression of both homoeologues of the precursors of *miR5200* (Table 4), a miRNA which represses *FT1* expression in SD in *Brachypodium* (Fig. 4b) [61]. Increased levels of miR5200 in the *phyC*-null mutant may contribute to the very low expression of *FT1* in these plants.

Finally, we identified 23 transcripts annotated as repetitive elements among the high-confidence *PHYB*-regulated genes and 11 repetitive elements among the *PHYC*-regulated genes (Additional file 3). Induced expression of wheat repetitive elements has been observed under stressful conditions [62], but the effect of these changes is currently unknown. Differentially regulated repetitive elements in this study include both DNA transposons and retrotransposons, and the majority were upregulated in the *phy-*null mutant relative to the wild-type (65 and 91 % were upregulated in the *phyB*-null mutant and *phyC*-null mutant, respectively). No repetitive elements were identified among the 82 differentially expressed genes regulated in a concerted manner by both PHYB and PHYC proteins.

## Discussion

Phytochromes are ubiquitous among photosynthetic eukaryotes and, within flowering plants, share a highly conserved protein structure [13]. However, despite their similarities at the protein level, differences in phytochrome function have been reported among different species of flowering plants. For example, in both rice and Arabidopsis the PHYC protein is unstable in the absence of other phytochromes, whereas the wheat PHYC protein is stable and functional when transformed into an Arabidopsis mutant lacking all phytochromes [19]. This suggests that in wheat, the PHYB and PHYC proteins can act both independently and in concert to control downstream regulatory pathways.

To characterize genes regulated by PHYB and PHYC in wheat, we applied a highly stringent approach using replicated RNA-seq studies. A FDR-adj *P* <0.01 was used for each experiment and only those genes that were significant in both experiments were defined as high-confidence genes. Since the two experiments were independent, the high-confidence genes have an FDR < 0.0001, resulting in less than one expected false positive in each selected set (PHYB = 0.04 and PHYB = 0.23). This stringent criterion is likely to exclude many genuine PHY-regulated genes (false negatives), so users of this data are encouraged to analyze the data using different levels of stringency (Additional file 2). The subset of FDR-adj *P* <0.01 PHYB- and PHYC-regulated genes significant in just one experiment (Additional file 4) likely includes additional true positives which may provide valuable insight into the PHY-mediated regulation of light signaling. Currently, many of the differentially expressed genes identified in this study lack annotation, indicating that additional research will be required to determine their function and their role in the transduction of light signals in wheat.

### Potential mechanisms for the concerted action of *PHYB* and *PHYC*

**Fig. 3:**
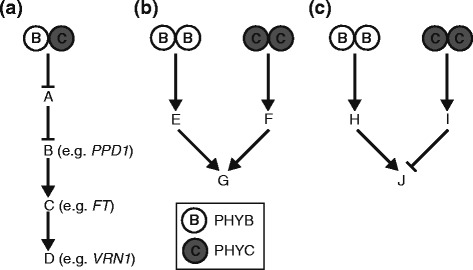
Potential mechanisms of concerted regulation of downstream genes by PHYB and PHYC. **a** Heterodimer regulation: regulation by a PHYB/PHYC heterodimer results always in changes in the same direction in *phyB*-null and *phyC*-null mutants. **b-c** Homodimer regulation: independent regulation by PHYB/PHYB and PHYC/PHYC homodimers can result in changes in the same or opposite direction. **b** Gene “G” is differentially regulated in the same direction in both *phyB*-null and *phyC*-null mutants**. c** Gene “J’ is upregulated in the *phyB-*null and downregulated in *phyC*-null mutant

We identified 104 genes that were differentially regulated in both *phyB-*null and *phyC*-null mutants, suggesting that PHYB and PHYC can act in a concerted manner to regulate a small subset of target genes. Figure 3 presents alternative mechanisms to explain this concerted action. If a gene/protein is regulated by the PHYB-PHYC heterodimer, loss-of-function mutations in either of these phytochromes will result in similar changes in the expression (either induction or repression) of the target gene and all its downstream targets (Fig. 3a). A similar concerted effect will also be observed if PHYB-PHYB and PHYC-PHYC homodimers each regulate the same transcription factor independently, or alternatively, regulate separate transcription factors that have similar effects on the regulation of a third gene (Fig. 3b). If the PHYB-PHYB and PHYC-PHYC homodimers regulate genes that have opposite effects on the regulation of the target gene, the *phyB*-null and *phyC*-null mutants will exhibit changes in the expression of this gene in opposing directions (Fig. 3c). This last mechanism can explain the 22 high-confidence genes that were differentially regulated in the *phyB*-null and *phyC*-null mutants in the opposite direction (Additional file 3). These regulatory pathways are likely to be more complex than the simplified mechanisms presented in Fig. 3, as some genes may be regulated by both homo- and hetero-PHY dimers. This model is presented only to show that multiple mechanisms can generate similar concerted effects.

In this transcriptome analysis more than 95 % of the high-confidence genes showed significant differences in expression either in the *phyB*-null or *phyC*-null mutants alone (Fig. 2c). Genes regulated by PHYB-PHYB or PHYB-PHYA dimers are likely to be included in the set of genes differentially expressed only in the *phyB*-null mutant, whereas those regulated by PHYC-PHYC or PHYC-PHYA dimers are likely to be included in the set of genes differentially expressed only in the *phyC*-null mutant. However, these two sets also include genes that are regulated in the same direction in both mutants, but that are highly-significant (significant in both experiments) only for one of the phytochrome mutants.

### Differences in phytochrome function among flowering plants

Our study also revealed variable functions of the phytochromes in the regulation of flowering between monocot and dicot flowering plants. Whereas loss-of-function mutations in *PHYB* are associated with early flowering in Arabidopsis [63], in wheat they are associated with an extreme delay in flowering (Fig. 1b), despite both species exhibiting accelerated flowering in a LD photoperiod. In addition, loss-of-function mutations in *PHYC* have a limited effect on Arabidopsis flowering under LD [17, 64], but result in extreme late flowering in wheat [19], barley [20] and *Brachypodium* [21].

**Fig. 4:**
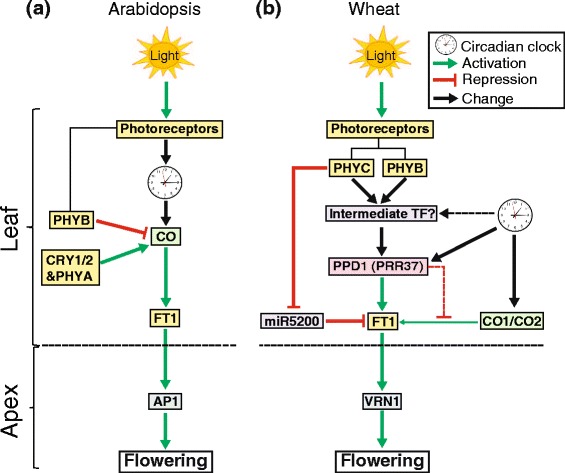
Simplified models of photoperiodic regulation of flowering in (**a**) Arabidopsis (LD dicot species) based on Valverde et al. [65] and (**b**) wheat (LD monocot species) based on Chen and Dubcovsky 2012 [87] and Chen et al. 2014 [19]. In Arabidopsis the photoperiodic response is regulated by CONSTANS (CO). In the absence of PHYB, CO proteins accumulate, inducing flowering. In wheat, PPD1/PRR37 is the central regulator of the photoperiodic response. The effects of CO in monocots are observed only in the absence of PPD1/PRR37 [88, 89]. In wheat, *PHYB* and *PHYC *are required for the light activation of PPD1/PRR37 so both the *phyB-*null and *phyC-null * mutants exhibit a late-flowering phenotype

These contrasting effects are more likely due to changes downstream of the phytochrome signaling pathway that occurred during the divergence of monocots and dicots than to changes in the phytochrome proteins. In Arabidopsis, LD acceleration of flowering is mediated by CONSTANS (CO). Since PHYB reduces CO abundance during the morning [65] (Fig. 4a), *phyB* loss-of-function mutations in Arabidopsis result in the accumulation of CO and the subsequent activation of FT and accelerated flowering (Fig. 4a). By contrast, in the temperate grasses the acceleration of flowering under LD is mainly mediated by PPD1/PRR37, a Pseudo Response Regulator that originated from a gene duplication in the grass lineage that originated *PRR37* and *PRR73*, and that was independent of the event that originated the *PRR3* and *PRR7* genes in Arabidopsis [66] (Fig. 4b). The sub-functionalization of the duplicated *PPD1/PRR37* as a photoperiod gene in the grass lineage is not observed in the corresponding Arabidopsis *PRR3* or *PRR7* genes. In wheat, because both PHYB (Fig. 1b) and PHYC [19] are required for the light activation of *PPD1*, and its downstream target FT1, loss-of-function mutations in either phytochrome result in extreme delays in flowering under LD, despite the upregulation of *CO1* in these mutants [19] (Fig. 4b).

### Additional effects of *PHYB* and *PHYC* on flowering induction in wheat

Although the *PPD1* gene plays a central role in the regulation of the photoperiod response in wheat, the strong downregulation of *PPD1* in the *phyB*-null (120-fold reduction) and *phyC-*null mutants (30-fold reduction) is not sufficient to explain the drastic delay in flowering time observed in these mutants in LD photoperiods (Fig. 1b, [19]). The combined loss-of-function of the three homoeologues of *PPD1* in the hexaploid wheat variety Paragon delays flowering by only one month, whereas the *phyB*-null and *phyC*-null mutants exhibit a flowering delay of more than three months. This suggests that PHYB and PHYC also regulate other flowering genes. Additional genes associated with flowering time identified in our study include positive (e.g., *GATA* transcription factors, *VIN3*) and negative (e.g., *TaAGL33*) regulators of flowering. Of the six uncharacterized members of the PEBP family found in our study, five were more highly expressed in the late-flowering *phyB*-null mutant than in the wild type (Table 3). Some PEBP proteins have been shown to act as floral repressors in other species [67], so it is possible that these upregulated *FT-*like genes contribute to the late flowering phenotype of the *phyB-*null mutant. Alternatively, they may be part of a feedback loop that compensates for the lack of *FT1* expression and are actually favoring the eventual late flowering of the *phyB*-null mutant. It would be interesting to characterize loss-of-function mutants for these genes to understand their specific role in regulating flowering time in wheat. We also identified several differentially expressed genes which encode components of the circadian clock (e.g., *LHY, CCA1, GI* and *PRR95*). In Arabidopsis, approximately one-third of all genes are transcriptionally regulated by the circadian clock [68], suggesting that the changes generated in the core clock genes in the *phyB-*null (Table 3) and the *phyC-*null mutant [19] may result in large-scale changes in gene expression that contribute to the large delay in flowering time observed in these mutants. In the *phyC-*null mutant, the upregulation of *miR5200* and the post-transcriptional downregulation of *FT1* [61] may also contribute to the late flowering phenotype of this mutant (Fig. 4b).

### *PHYB* and the shade-avoidance response in wheat

Observations from our phenotypic and transcriptomic studies suggest that *PHYC* likely plays a narrower role than *PHYB* in wheat development. Although characterized by delayed flowering and changes in spikelet and floret morphology (e.g., a reduced number of florets and elongated rachillas, glumes, and awns), the *phyC*-null mutant still produces normal flowers and seeds [19]. The strong effect on flowering time and spike morphology, together with the known role in flowering regulation of many of the genes differentially regulated in the *phyC*-null mutant suggest that a central role of *PHYC* is to regulate flowering time and spike development in wheat. However, the longer and narrower leaves found in the *phyC*-null mutant indicate that this gene also affects other developmental processes.

**Fig. 5:**
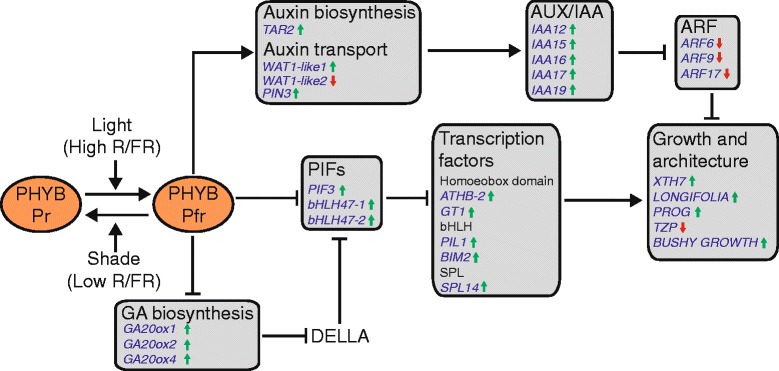
Putative members of the shade-avoidance response pathway in wheat. Genes significantly up (⬆) or down (⬇) regulated in the *phyB*-null mutant are displayed. Gene names are in blue

The *phyB*-null mutant plants flower later, are sterile and exhibit altered vegetative development (e.g., elongation of the internodes between tillers, longer and wider leaves and an increased rate of leaf production) (Fig. 1c, Additional file 1: Figure S2). In agreement with the phenotypic differences between mutants we found that the *phyB***-**null mutant exhibited differential expression of a larger and more diverse set of genes than the *phyC***-**null mutant. For example, a number of auxin, gibberellin, brassinosteroid and ethylene biosynthesis and signaling genes are differentially expressed in the *phyB*-null mutant, all hormones which are important regulatory components of the shade-avoidance response [39, 69–71] (Table 3 and Fig. 5). In addition, the *phyB*-null mutant showed expression changes in the wheat orthologues of several shade-avoidance regulatory genes and in genes with putative roles in regulating cell elongation and architecture (Table 3 and Fig. 5) [48–50, 55].

Experiments controlling R/FR ratios to simulate shaded conditions in dense stands found that wheat yields, even in modern varieties, were reduced in light conditions of low R/FR ratios [72]. Since the low R/FR ratios used in these experiments did not promote stem or leaf sheath growth, the negative effects on yield were attributed to delayed spike development and reduced floret number [72]. Low R/FR ratios favors formation of the inactive Pr form of the phytochromes, so the delayed flowering under these conditions is consistent with the observed delayed flowering in the loss-of function *phyB*-null and *phyC*-null mutants. By contrast, in Arabidopsis and other dicot species, low R/FR light ratios (or *phyB* mutations) result in accelerated reproductive development, a characteristic of the shade-avoidance response [15]. To better characterize the impact of the shade avoidance response in wheat, it will be necessary to separate the effects of low R/FR light on flowering from the effects it has on vegetative development by dissecting the complex transcriptional networks controlled by *PHYB*.

Further studies will also be required to investigate the links between PHYB-mediated light signaling and tolerance to abiotic stresses such as cold and drought. The wheat *phyB*-null mutant exhibits a significant reduction in the expression of two ABA receptors, a transcriptional profile which, in Arabidopsis, is indicative of reduced ABA-sensitivity and lower drought tolerance through effects on stomatal conductance [73]. *PHYB* also activates members of the cold acclimation pathway in response to low R/FR light in Arabidopsis [52]. The principal targets during this response are members of the *C-REPEAT/DRE BINDING FACTOR* (*CBF*) family of transcriptional regulators. *CBF* genes activate downstream *COR* genes which play a functional role protecting the cell against frost and desiccation damage [52]. While we did not detect any differentially-expressed *CBF* genes within our dataset, four wheat *COR* genes were upregulated in the *phyB*-null mutant and one member of the *INDUCER of CBF EXPRESSION* (*ICE*) gene family [53] was downregulated (Table 3), suggesting that the role of *PHYB* in the light-mediated activation of the cold acclimation pathway observed in Arabidopsis [52] may be conserved in wheat.

### Effect of *PHYB* and *PHYC* in the regulation of repetitive elements and miRNAs

In addition to their effect on the expression of hundreds of protein-coding genes, both the *phyB-*null and *phyC-*null mutants exhibit differential expression of several repetitive elements. This suggests a possible role of the phytochromes (or more likely of its downstream targets) in the transcriptional regulation of such elements.

In addition to directly affecting the rate of transcription, gene activity may also be impacted post-transcriptionally through the activity of miRNAs, a class of small ncRNAs which target specific mRNA transcripts for cleavage and thus inactivation [74]. Both *phy-*null mutants showed high-confidence differentially expressed miRNAs. The upregulation of pri-*miR5200* in the *phyC*-null mutant relative to the wild-type is consistent with the increased levels of *miR5200* observed under SD than under LD in *Brachypodium* [61]. Since *phyC*-null plants cannot perceive the LD signal [19], *miR5200* levels are maintained at high levels even under LD, whereas they are greatly reduced in these conditions in wild-type wheat and *Brachypodium* plants [61]. The post-transcriptional downregulation of the central wheat flowering promoter *FT1* by *miR5200* may contribute to the extremely late flowering of the *phyC*-null mutant. The *phyB*-null mutant exhibited increased expression of pri-*miR156* (Table 4), which has been associated with a prolonged vegetative state in maize [75] and switchgrass [76], consistent with the phenotype of the wheat *phyB*-null mutant.

## Conclusion and future directions

This study demonstrates that both PHYB and PHYC are required for the acceleration of wheat flowering under LD, an effect which is partially mediated by the transcriptional activation of *PPD1*. Both *phyB*-null and *phyC-*null mutants exhibit changes in the expression of circadian clock genes, and their disruption may contribute to the dramatic flowering delay observed in these plants. In addition, this study revealed that PHYB and PHYC also specifically regulate certain pathways. PHYC activity, but not PHYB is required for the downregulation of *miR5200*, a post-transcriptional repressor of the flowering promoter *FT1*. PHYB is actively involved in the regulation of the shade-avoidance response. In contrast with Arabidopsis and the SD grasses, *phyB*-null mutations in wheat (as well as reduced R/FR ratios) result in delayed flowering. Therefore, a modification of wheat responses to growth in dense stands will require the separation of the effects of PHYB on flowering from its effects on other components of the shade-avoidance response. The PIFs are excellent candidates to initiate the dissection of these pathways since they interact directly with the phytochromes and are critical hubs that integrate temperature, light and hormonal signals to regulate development [6, 77]. RNA-seq experiments using different combinations of wheat *pif-*null mutants may help to dissect the complex *PHYB/PHYC* effects described in this study.

## Methods

### Plant materials

We identified *phyB*-null mutant lines by screening a TILLING population in the tetraploid *Triticum turgidum* L*. subsp.* durum (Desf.) var. Kronos using a protocol described previously [78]. Full-length genomic sequences of the *PHYB-A* and *PHYB-B* homoeologues were identified from the draft genome assemblies of *Triticum urartu* [79] and *Aegilops speltoides*, respectively, and used to design homoeologue-specific primers to amplify fragments of each gene in ‘Kronos’. PCR-amplification of specific fragments were performed using the following conditions – 95 °C for 5 m, 40 cycles of: 95 °C for 30 s, 62/66 °C for 30 s, 72 °C 1 min/kb; 72 °C 7 min (annealing temperatures were 62 °C and 66 °C for the *PHYB-A* and *PHYB-B* TILLING fragments, respectively). Mutations in *PHYB-A* were detected by *Cel*Ι digestion of a 1875 bp PCR product amplified using the primers PHYB-A-F1 (5’-CTCTCCATCGCTGACGCAGTT-3’) and PHYB-A-R1 (5’-GATTGCTCTGACCCAAATGTCTTC-3’), and mutations in *PHYB-B* were identified by digesting a 1007 bp PCR fragment amplified with primers PHYB-B-F1 (5’-CCATGTTTGCAGATGTTGCAG-3’) and PHYB-B-R2 (5’-AGGTGTACATCCAGTCAGGTTGCA-3’). A CAPS marker was developed to genotype the *phyB-B* mutation by digesting the amplified TILLING product with the restriction enzyme *Hpy*Ch4V and running products on a 3 % polyacrylamide gel stained with ethidium bromide. After digestion with this enzyme the mutant allele shows a 407 bp band and the wild-type allele shows two fragments of 224 bp and 183 bp. The *phyB-A* mutation was detected by Sanger sequencing using the sequencing primer 5’-ATATCATCGAGTGGTTGACG-3’. The selected M_3_ lines carrying *phyB-A* and *phyB-B* null mutations were each backcrossed twice to wild-type ‘Kronos’ to reduce the impact of background mutations, before combining them to generate a *phyB*-null mutant line. Wild-type BC_2_F_2_ sister lines from these crosses were used as control plants in each experiment. Because *phyB*-null plants were sterile, backcrosses and F_2_ seed production were performed maintaining one of the mutations in a heterozygous state. Null mutations for *PHYC* were described previously [19]. The source of all plant materials was UC Davis.

### Growth conditions

All plants were grown in PGR15 growth chambers (Conviron, Manitoba, Canada) under LD conditions (16 h light/8 h dark) at 20 °C day/18 °C night temperatures and a light intensity of ~260 μM m^−2^ s^−1^. All chambers used similar halide light configurations and were located in the same room. PHYB and PHYC experiments were run separately. The two replications of the PHYB experiment were performed one after the other in the same chamber, but the two replications of the PHYC experiment were performed in separate chambers that we later realized had different ballast systems. The light intensity in each growth chamber at R (655-665 nm) and FR (725-735 nm) wavelengths was measured using a FieldSpec® HandHeld 2 visible near-infra-red Spectroradiometer (ASN Inc., Boulder Colorado). All statistical comparisons between mutant and wild-type controls are made within the same chamber so they are unaffected by variation between chambers.

### RNA-seq library construction and sequencing

The fully extended 3^rd^ leaf of four-week-old plants was harvested 4 h after the beginning of a 16 h light period (LD) for each genotype and stored immediately in liquid nitrogen. For each experiment, four biological replicates were used. Leaf tissues were ground into a fine powder in liquid nitrogen and total RNA was extracted using the Spectrum™ Plant Total RNA kit (Sigma-Aldrich, St. Louis, MO). Sequencing libraries were produced using the TruSeq RNA Sample Preparation kit v2 (Illumina, San Diego, CA), according to the manufacturer’s instructions. Library quality was determined using a high-sensitivity DNA chip run on a 2100 Bioanalyzer (Agilent Technologies, Santa Clara, CA). Libraries were barcoded to allow multiplexing within a single lane and were sequenced using the 50 bp SE module on a HiSeq2000 sequencer at the UC Davis Genome Center.

Raw reads were processed using a pipeline incorporating “*Scythe*” (https://github.com/vsbuffalo/scythe) to remove Illumina adapter contamination (Default options) and “*Sickle*” (https://github.com/najoshi/sickle) to remove low-quality reads (Default options except –l 25 –q 25). Trimmed reads were mapped to the A and B chromosome arms from the latest version of the draft wheat genome assembly in the hexaploid variety Chinese Spring (v2.2) from the IWGSC [31]. RNA-seq reads were mapped using GSNAPl, a splicing-aware aligner (version 05-09-2013, default parameters except -m 2 -n 1 -N 1 -A sam [80]) to generate Sequence Alignment/Map (SAM) files for each sample.

To define regions corresponding to transcribed gene-coding regions within this reference assembly, we performed a similar analysis as described previously [81]. Briefly, a non-redundant set of wheat transcripts from several transcriptomes were mapped to the A and B chromosome arm assemblies separately using GMAP (Version 05-09-2013), default parameters except -n 1 --nofails --cross-species -f samse -x 0 [82]. *Bedtools cluster* (-d 0) was then used to merge overlapping aligned regions, followed by *bedtools merge* to merge overlapping regions into a single putative transcribed region. The resulting General Feature Format (GFF) file consisted of 150,754 genomic ranges, each representing the genomic contig identifier and the start and end coordinate of the putative transcribed region.

Raw count values were generated using *ht-seq count* (-m union) using the generated GFF file and individual Sequence Alignment/Mapping (SAM) files for each sample. All reads with a mapping quality (MAPQ) value less than 40 from the SAM file (signifying an ambiguous, non-unique mapping position), were discarded at this stage, ensuring that expression values were generated using only uniquely-mapped reads. This approach generates homoeologue-specific expression profiles [32]. The percent of uniquely-mapped reads in each sample is described in Additional file 1: Table S1.

We used a custom ‘R’ package ‘*noleaven*’ (https://github.com/topherconley/noleaven) to remove contigs which had zero or very low numbers of counts. For each experimental replication, contigs which had less than three reads mapping to at least two biological replicates in the experiment were removed.

Raw counts were normalized using *DESeq* (Version 1.12.1 [83], R Version 2.14.2). After normalization, we applied the statistical tests implemented in both *DESeq* and *edgeR* [84] to classify differentially expressed genes in pair-wise comparisons. The *P*-values generated by both analyses were adjusted for FDR, using the procedure of Benjamini and Hochberg [85] and we selected a stringent cutoff of adjusted *P* ≤0.01 for significance for both tests within each experimental replication. Throughout the paper, both *DESeq* and *edgeR* results are presented as FDR-adjusted *P* values. We then selected for further analyses those genes that were significant in both replications under the criteria outlined above, and designated those genes as “high-confidence” differentially expressed genes (FDR < 0.0001).

### Functional annotation

For functional annotation, we identified the longest transcribed contig mapping to each genomic locus and performed a BLASTX against the nr protein database (nr.28, Apr 24, 2015 release, NCBI) and a BLASTP using the translated ORF against the Pfam database version 27.0 with InterProScan version 5.13 to identify conserved protein domains. The output was used to infer GO terms associated with each genomic locus using BLAST2GO version 2.6.5 and used the ‘R’ package *TopGO* version 2.14.0 to perform an enrichment analysis among the differentially regulated gene sets. “Biological Process” terms were obtained and significance values for enrichment were calculated using ‘classic’ Fishers’ exact test, as implemented in *TopGO*. Wheat miRNAs were annotated based on the closest rice homologue identified from the miRNA database “miRBase” (Release 21, [86]), except for miR5200, which was identified and annotated using the Brachypodium homologues [61].

### *qRT-PCR* validation of flowering time genes

The most recently-emerged leaf from wild-type and *phyB*-null plants were collected four hours after the beginning of a 16-h photoperiod from 2-week, 4-week and 6-week old plants. Harvested tissue was ground to a fine powder in liquid nitrogen and RNA was extracted as described above for RNA-seq library preparation. cDNA was synthesized using the High Capacity Reverse Transcription Kit (Applied Biosystems, Foster City, CA) according to the manufacturer’s instructions. Quantitative RT-PCR was performed using SYBR Green and a 7500 Fast Real-Time PCR system (Applied Biosystems, Foster City, CA). Primers for the target genes *PPD1, CO1*, *FT1*, *FT2*, *VRN1*, *VRN2* and the control gene *ACTIN* were described previously [19, 27]. Expression data are presented as fold-*ACTIN* levels.

## Abbreviations

ABA, Abscisic Acid; bHLH, basic Helix Loop Helix; BR, Brassinosteroid; FDR, False Discovery Rate; FR, Far red; GA, Gibberellin; GAF, cGMP phosphodiesterase/adenylate cyclase/FhlA; GFF, General Feature Format; LD, Long Day; MAPQ, Mapping Quality; PAS, period, aryl hydrocarbon receptor nuclear transporter and single minded; PEBP, Phosphatidylethanolamine-Binding Proteins; PHY, Phytochrome; PIF, Phytochrome Interacting Factor; R, Red; SAM, Sequence Alignment/Map; SD, Short Day; TILLING, Targeting Induced Local Lesions IN Genomes

## Additional files

Additional file 1: Table S1. Summary of RNA-seq reads and mapping. **Table S2**: TopGO analysis for functional enrichment for genes regulated by *PHYB*, *PHYC* and in concert by both *PHYB* and *PHYC*. **Figure S1.** Spike and floral organ phenotype of the *phyB*-null mutant. (a) Whole spike, (b) Single spikelet (c) Separated spikelet and (d) stamen and stigma. (e) Comparison of internode length between 83-day-old *phyB*-null, *phyC*-null and wild-type Kronos plants. Leaves have been removed to facilitate visualization of internodes. Nodes are indicated by purple arrows. Bar = 10 cm. **Figure S2.** Vegetative phenotype of wild-type control, *phyB*-null and *phyC*-null plants. (a) Leaf emergence rate, (b) leaf length and (c) width at three different timepoints. * *P* <0.05; ** *P* <0.01. **Figure S3.** Phenotype of four-week old *PHYB* wild-type, *phyB*-null, *PHYC* wild-type and *phyC*-null plants at the stage of harvest for RNA-seq analysis. **Figure S4.** Principal Component Analysis of normalized expression values of all genes. (a) All libraries, (b) PHYB libraries and (c) PHYC libraries. **Figure S5.** Relative transcript levels of six flowering time genes determined by qRT-PCR in wild-type and *phyB*-null mutants at three stages of development (Leaves from two-week, four-week and six-week-old plants). Expression levels are presented as fold-*ACTIN*. * *P* < 0.05, ** *P* < 0.01, *** *P* < 0.001. (PDF 537 kb) Additional file 2: All RNA-seq data. Columns describe the genomic locus, the associated longest transcript which mapped to this locus, top BLAST hit and description and e-value, normalized counts from each library, fold-change (wild-type/*phy-*null mutant, genes highlighted green are upregulated in the *phy*-null mutant, genes highlighted red are downregulated in the *phy*-null mutant) and FDR-adjusted *P* values from DESeq and EdgeR (*P*adj <0.01 highlighted in yellow). (XLSX 60849 kb) Additional file 3: High-confidence PHYB/PHYC-regulated genes. In different tabs, 2202 PHYB-regulated genes, 261 PHYC-regulated genes, 82 genes showing concerted regulation by PHYB and PHYC in the same direction and 22 genes showing concerted regulation by PHYB and PHYC in the opposite direction. Columns describe the genomic locus, the associated longest transcript which mapped to this locus, top BLAST hit and description and e-value, normalized counts from each library, fold-change (wild-type/*phy-*null mutant, genes highlighted green are upregulated in the *phy*-null mutant, genes highlighted red are downregulated in the *phy*-null mutant) and FDR-adjusted *P* values from DESeq and EdgeR (*P*adj <0.01 highlighted in yellow). (XLSX 1200 kb) Additional file 4: FDR-adj *P* <0.01 PHYB/PHYC-regulated genes significant in just one experiment. In different tabs, 5320, PHYB-regulated genes, 1037 PHYC-regulated genes and 336 genes showing concerted regulation by PHYB and PHYC. Columns describe the genomic locus, the associated longest transcript which mapped to this locus, top BLAST hit and description and e-value, normalized counts from each library, fold-change (wild-type/*phy-*null mutant, genes highlighted green are upregulated in the *phy*-null mutant, genes highlighted red are downregulated in the *phy*-null mutant) and FDR-adjusted *P* values from DESeq and EdgeR (*P*adj <0.01 highlighted in yellow). (XLSX 3100 kb)
