# Supplementary material for: RNA-seq studies using wheat PHYTOCHROME B and PHYTOCHROME C mutants reveal shared and specific functions in the regulation of flowering and shade-avoidance pathways
Source: BMC Plant Biol. 2016 Jun 21;16:141. doi: 10.1186/s12870-016-0831-3 (PMC4915087; doi:10.1186/s12870-016-0831-3)

**RNA-seq studies using wheat *PHYTOCHROME B* and *PHYTOCHROME C* mutants reveal shared and specific functions in the regulation of flowering and shade-avoidance pathways**

**Additional file 1**

Tables and figures in this additional file:

**Table S1:** Summary of RNA-seq reads and mapping.

**Table S2:** TopGO analysis for functional enrichment for genes regulated by *PHYB*, *PHYC* and in concert by both *PHYB* and *PHYC*.

**Figure S1:** Spike and floral organ phenotype of the *phyB*-null mutant. (a) Whole spike, (b) Single spikelet (c) Separated spikelet and (d) Stamen and stigma. (e) Comparison of internode length between 83-day-old *phyB*-null, *phyC*-null and wild-type Kronos plants. Leaves have been removed to facilitate visualization of internodes. Nodes are indicated by purple arrows. Bar = 10 cm.

**Figure S2:** Vegetative phenotype of wild-type control, *phyB*-null and *phyC*-null plants. (a) Leaf emergence rate, (b) Leaf length and (c) leaf width at three different timepoints (* $P$ <0.05; ** $P$ <0.01).

**Figure S3:** Phenotype of four-week old *PHYB* wild-type, *phyB*-null, *PHYC* wild-type and *phyC*-null plants at the stage of harvest for RNA-seq analysis.

**Figure S4:** PCA plots of normalized expression values of all genes. (a) All libraries, (b) PHYB libraries and (c) PHYC libraries.

**Figure S5:** Relative transcript levels of six flowering time genes determined by qRT-PCR in wild-type and *phyB*-null mutants at three stages of development (Leaves from two-week, four-week and six-week-old plants). Expression levels are presented as fold-*ACTIN*.

* $P$ < 0.05, ** $P$ < 0.01, *** $P$ < 0.001.

**Table S1:** Summary of RNA-seq reads and mapping rates.

| Genotype | Experimental replicate | Biological replicate | Raw reads | Trimmed reads | % mapped to gene loci | % uniquely mapped to gene loci |
|---|---|---|---|---|---|---|
| ***PHYB* wild-type** | 1 | 1 | 42,783,726 | 38,193,836 | 95.6 | 58.0 |
| | | 2 | 42,254,752 | 37,228,077 | 95.4 | 58.1 |
| | | 3 | 58,349,947 | 50,793,083 | 95.6 | 58.1 |
| | | 4 | 50,148,260 | 44,301,543 | 95.9 | 56.5 |
| | 2 | 1 | 57,790,105 | 53,475,278 | 95.5 | 58.4 |
| | | 2 | 58,798,668 | 54,275,607 | 95.0 | 58.6 |
| | | 3 | 61,694,504 | 57,095,077 | 95.6 | 58.7 |
| | | 4 | 64,329,742 | 59,581,947 | 95.0 | 58.8 |
| ***phyB*-null** | 1 | 1 | 48,416,815 | 43,374,525 | 94.8 | 58.5 |
| | | 2 | 56,768,866 | 47,201,620 | 94.7 | 58.0 |
| | | 3 | 88,913,796 | 81,109,422 | 94.9 | 57.6 |
| | | 4 | 53,140,168 | 47,250,183 | 93.9 | 58.5 |
| | 2 | 1 | 51,385,316 | 47,550,092 | 95.0 | 59.1 |
| | | 2 | 54,947,620 | 50,880,434 | 94.7 | 59.2 |
| | | 3 | 60,875,008 | 56,371,576 | 94.6 | 59.0 |
| | | 4 | 55,147,736 | 51,113,770 | 94.7 | 59.1 |
| ***PHYC* wild-type** | 1 | 1 | 41,280,436 | 40,263,724 | 95.7 | 59.6 |
| | | 2 | 41,700,104 | 40,409,065 | 95.8 | 62.3 |
| | | 3 | 43,295,783 | 42,605,342 | 95.6 | 59.8 |
| | | 4 | 40,970,343 | 39,796,668 | 95.7 | 60.5 |
| | 2 | 1 | 38,880,169 | 37,994,979 | 95.4 | 60.3 |
| | | 2 | 44,203,882 | 43,262,009 | 95.5 | 59.6 |
| | | 3 | 42,528,948 | 41,619,486 | 95.4 | 59.7 |
| | | 4 | 32,593,169 | 31,887,538 | 95.4 | 59.8 |
| ***phyC*-null** | 1 | 1 | 45,693,558 | 44,904,496 | 95.1 | 58.6 |
| | | 2 | 47,618,087 | 46,776,974 | 95.2 | 59.3 |
| | | 3 | 44,675,515 | 43,960,996 | 95.4 | 59.5 |
| | | 4 | 43,530,452 | 42,873,364 | 95.4 | 59.2 |
| | 2 | 1 | 36,142,687 | 35,291,647 | 95.2 | 59.7 |
| | | 2 | 37,545,579 | 36,712,107 | 95.4 | 58.9 |
| | | 3 | 46,594,218 | 45,656,274 | 95.1 | 59.7 |
| | | 4 | 39,781,242 | 38,922,327 | 96.7 | 41.0 |
| | **Average** | | **49,149,350** | **46,022,908** | **95.3** | **58.5** |

**Table S2:** TopGO analysis for functional enrichment for genes regulated by *PHYB*, *PHYC* and in concert by both *PHYB* and *PHYC*.

| | Accession | Ontology | Annotated | Significant | Expected | *P* |
|---|---|---|---|---|---|---|
| ***PHYB*-regulated** | GO:0009755 | Hormone-mediated signaling pathway | 1913 | 85 | 45.04 | 2.20E-08 |
| | GO:0032870 | Cellular response to hormone stimulus | 1928 | 85 | 45.39 | 3.10E-08 |
| | GO:0071495 | Cellular response to endogenous stimulus | 1930 | 85 | 45.44 | 3.30E-08 |
| | GO:0009725 | Response to hormone stimulus | 2598 | 103 | 61.17 | 2.10E-07 |
| | GO:0009719 | Response to endogenous stimulus | 2672 | 104 | 62.91 | 4.20E-07 |
| | GO:0043269 | Regulation of ion transport | 185 | 18 | 4.36 | 4.60E-07 |
| | GO:0009642 | Response to light intensity | 337 | 24 | 7.93 | 2.00E-06 |
| | GO:0051049 | Regulation of transport | 208 | 18 | 4.9 | 2.50E-06 |
| | GO:0006468 | Protein phosphorylation | 4258 | 146 | 100.25 | 2.90E-06 |
| | GO:0071310 | Cellular response to organic substance | 2428 | 93 | 57.16 | 3.40E-06 |
| ***PHYC*-regulated** | GO:0048586 | Regulation of long-day photoperiodism, flowering | 12 | 2 | 0.03 | 0.00036 |
| | GO:2000028 | Regulation of photoperiodism, flowering | 12 | 2 | 0.03 | 0.00036 |
| | GO:0048574 | Long-day photoperiodism, flowering | 30 | 2 | 0.07 | 0.00233 |
| | GO:0048571 | Long-day photoperiodism | 38 | 2 | 0.09 | 0.00372 |
| | GO:0015693 | Magnesium ion transport | 44 | 2 | 0.1 | 0.00496 |
| | GO:0006355 | Regulation of transcription, DNA-dependent | 3561 | 16 | 8.46 | 0.01022 |
| | GO:2001141 | Regulation of RNA biosynthetic process | 3561 | 16 | 8.46 | 0.01022 |
| | GO:0051252 | Regulation of RNA metabolic process | 3587 | 16 | 8.53 | 0.0109 |
| | GO:0048552 | Regulation of metalloenzyme activity | 6 | 1 | 0.01 | 0.01418 |
| | GO:0048554 | Positive regulation of metalloenzyme activity | 6 | 1 | 0.01 | 0.01418 |
| ***PHYB-PHYC* regulated** | GO:0006355 | Regulation of transcription, DNA-dependent | 3561 | 10 | 2.19 | 3.30E-05 |
| | GO:2001141 | Regulation of RNA biosynthetic process | 3561 | 10 | 2.19 | 3.30E-05 |
| | GO:0051252 | Regulation of RNA metabolic process | 3587 | 10 | 2.2 | 3.50E-05 |
| | GO:0010556 | Regulation of macromolecule biosynthetic process | 3910 | 10 | 2.4 | 7.20E-05 |
| | GO:2000112 | Regulation of cellular macromolecule biosynthetic process | 3910 | 10 | 2.4 | 7.20E-05 |
| | GO:0019219 | Regulation of nucleobase-containing compound metabolic process | 3926 | 10 | 2.41 | 7.50E-05 |
| | GO:0031326 | Regulation of cellular biosynthetic process | 3950 | 10 | 2.43 | 7.90E-05 |
| | GO:0051171 | Regulation of nitrogen compound metabolic process | 3955 | 10 | 2.43 | 8.00E-05 |
| | GO:0009889 | Regulation of biosynthetic process | 4011 | 10 | 2.47 | 9.00E-05 |
| | GO:0006351 | Transcription, DNA-dependent | 4194 | 10 | 2.58 | 0.00013 |

**Figure S1:** Spike and floral organ phenotype of the *phyB*-null mutant. (a) Whole spike, (b) Single spikelet (c) Separated spikelet and (d) Stamen and stigma. (e) Comparison of internode length between 83-day-old *phyB*-null, *phyC*-null and wild-type Kronos plants. Leaves have been removed to facilitate visualization of internodes. Nodes are indicated by purple arrows. Bar = 10 cm.

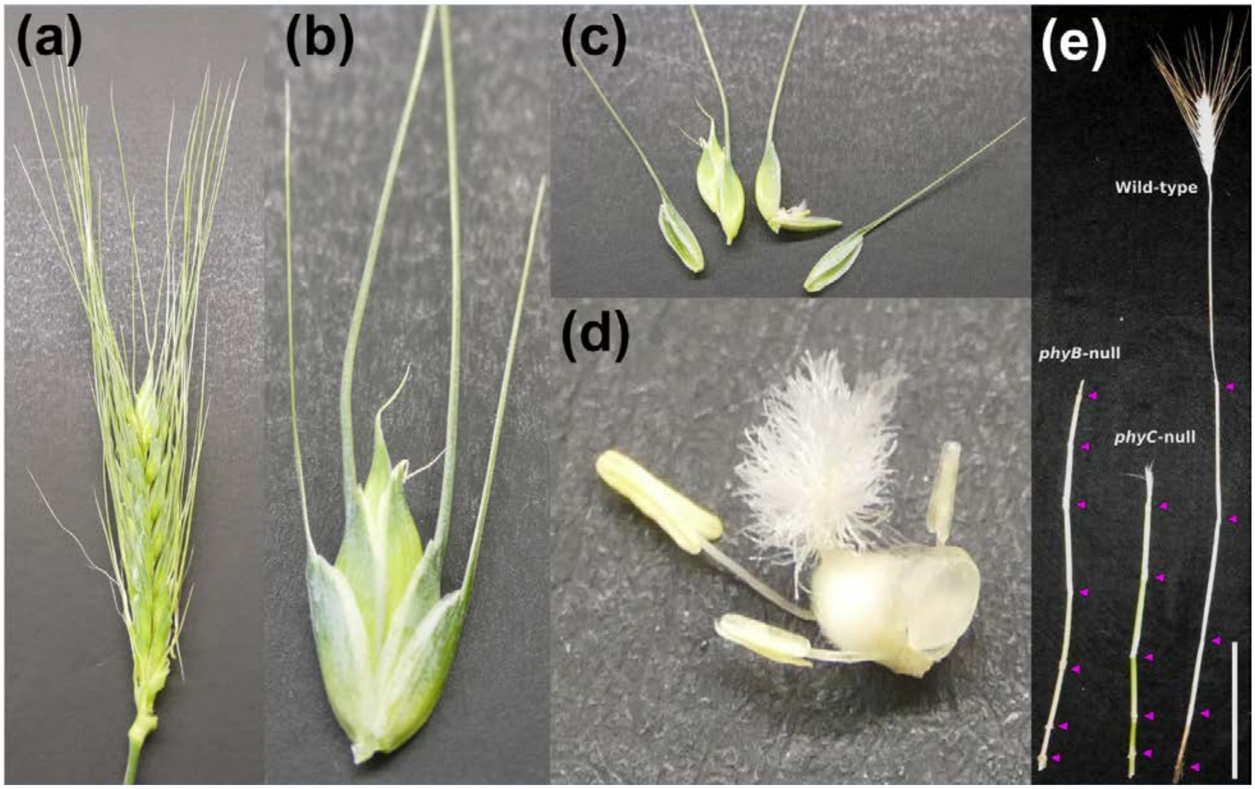

**Figure S2:** Vegetative phenotype of wild-type control, *phyB*-null and *phyC*-null plants. (a) Leaf emergence rate, (b) Leaf length and (c) leaf width at three different timepoints.

(* *P* <0.05; ** *P* <0.01).

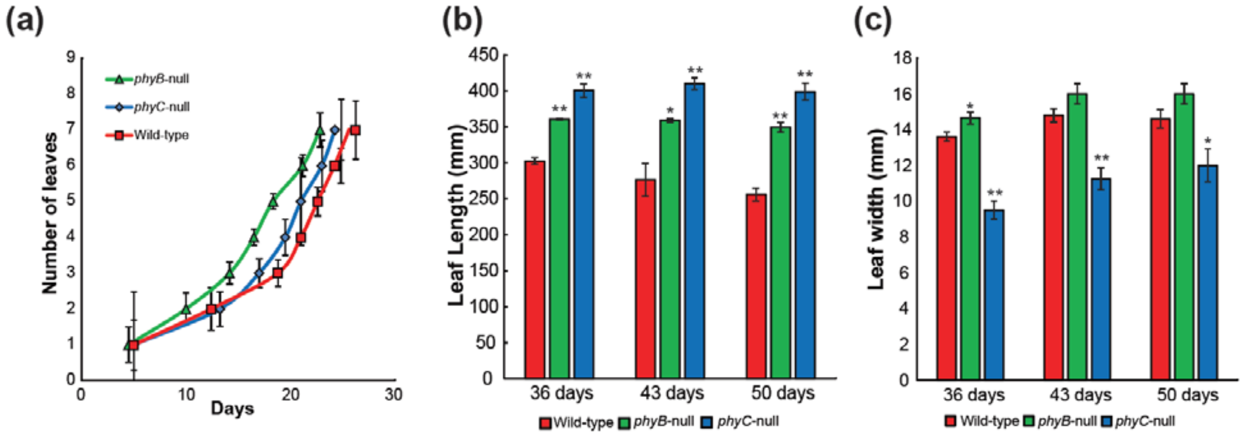

**Figure S3:** Phenotype of four-week old *PHYB* wild-type, *phyB*-null, *PHYC* wild-type and *phyC*-null plants at the stage of harvest for RNA-seq analysis.

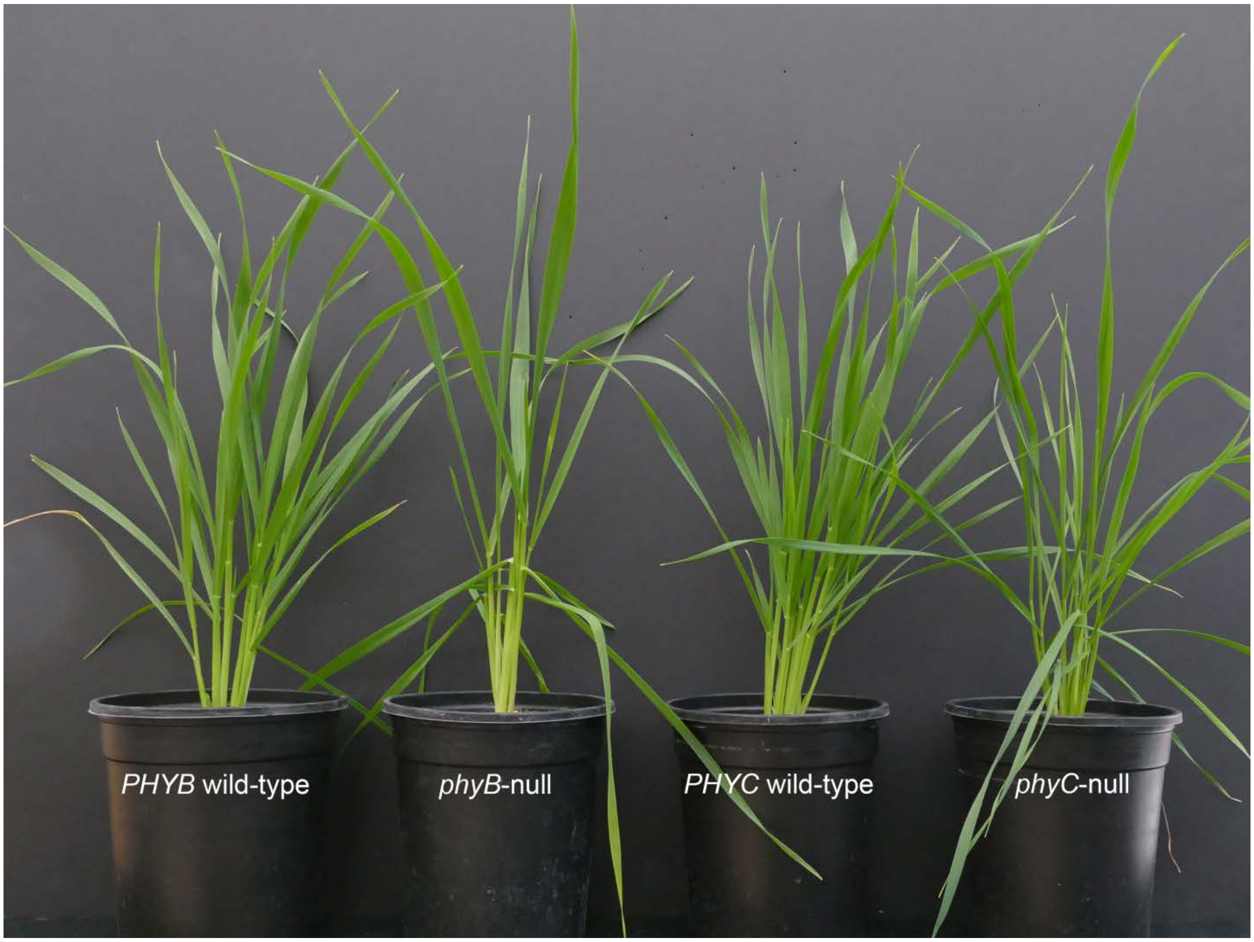

**Figure S4:** PCA plots of normalized expression values of all genes. (a) All libraries, (b) PHYB libraries and (c) PHYC libraries.

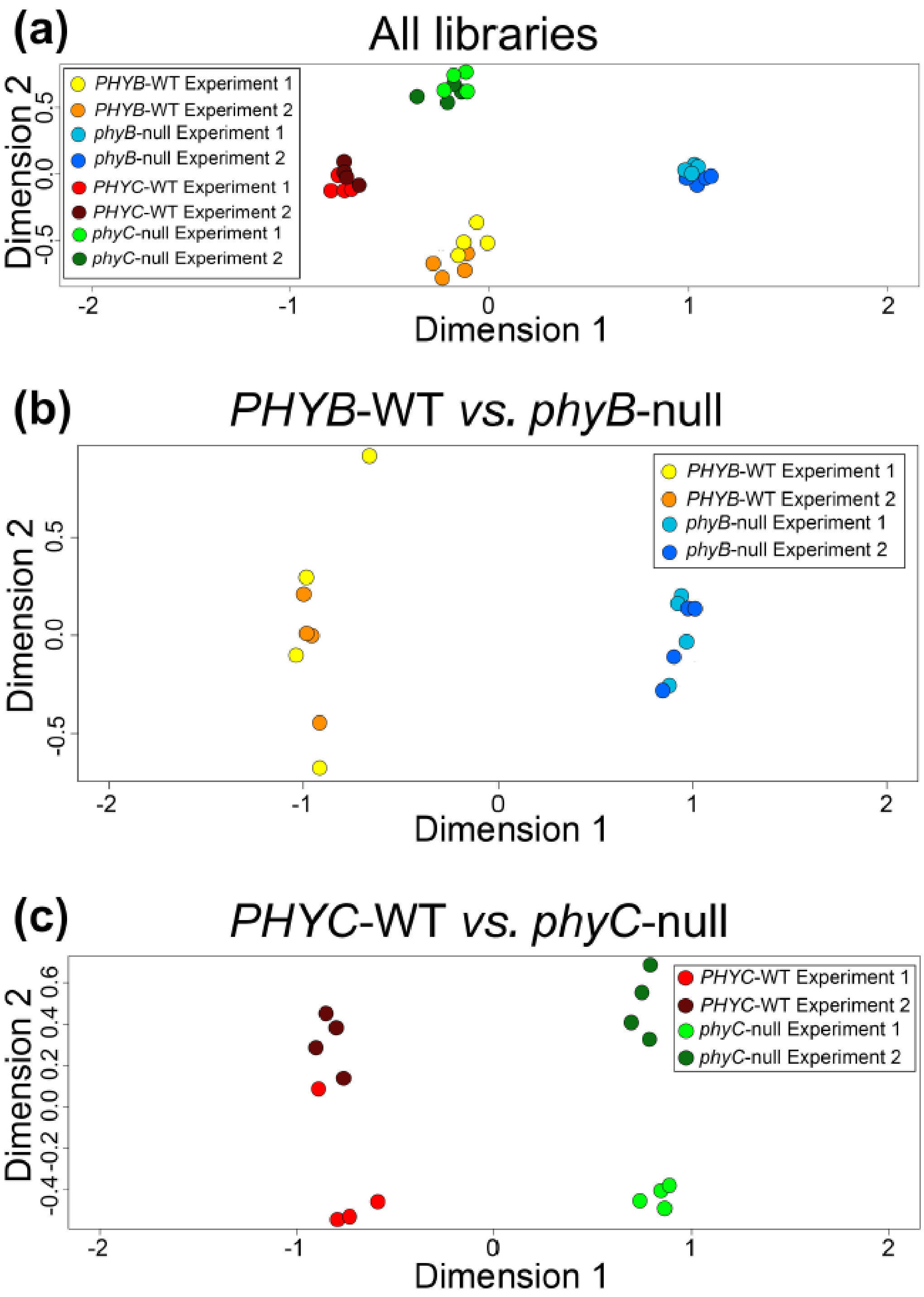

**Figure S5:** Relative transcript levels of six flowering time genes determined by qRT-PCR in wild-type and *phyB*-null mutants at three stages of development (Leaves from two-week, four-week and six-week-old plants). Expression levels are presented as fold-*ACTIN*.

* $P < 0.05$, ** $P < 0.01$, *** $P < 0.001$.

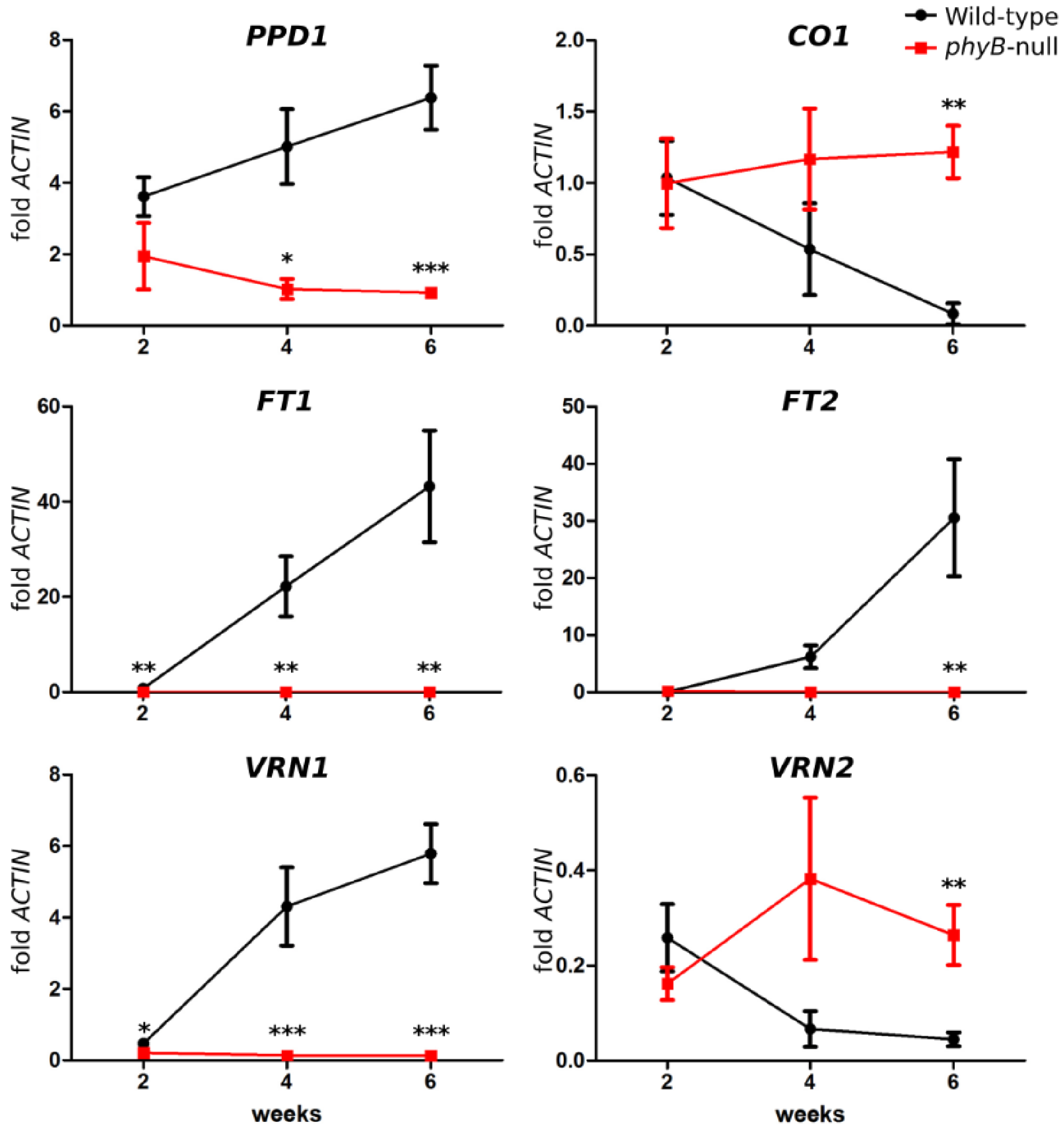

Supplement: Additional file 1: Table S1. — Summary of RNA-seq reads and mapping. Table S2: TopGO analysis for functional enrichment for genes regulated by PHYB, PHYC and in concert by both PHYB and PHYC. Figure S1. Spike and floral organ phenotype of the phyB-null mutant. (a) Whole spike, (b) Single spikelet (c) Separated spikelet and (d) stamen and stigma. (e) Comparison of internode length between 83-day-old phyB-null, phyC-null and wild-type Kronos plants. Leaves have been removed to facilitate visualization of internodes. Nodes are indicated by purple arrows. Bar = 10 cm. Figure S2. Vegetative phenotype of wild-type control, phyB-null and phyC-null plants. (a) Leaf emergence rate, (b) leaf length and (c) width at three different timepoints. * P <0.05; ** P <0.01. Figure S3. Phenotype of four-week old PHYB wild-type, phyB-null, PHYC wild-type and phyC-null plants at the stage of harvest for RNA-seq analysis. Figure S4. Principal Component Analysis of normalized expression values of all genes. (a) All libraries, (b) PHYB libraries and (c) PHYC libraries. Figure S5. Relative transcript levels of six flowering time genes determined by qRT-PCR in wild-type and phyB-null mutants at three stages of development (Leaves from two-week, four-week and six-week-old plants). Expression levels are presented as fold-ACTIN. * P < 0.05, ** P < 0.01, *** P < 0.001. (PDF 537 kb) [file 12870_2016_831_MOESM1_ESM.pdf]
